# "Test, Listen, Cure" (TLC) Hepatitis C Community Awareness Campaign

**DOI:** 10.2196/resprot.3822

**Published:** 2015-02-06

**Authors:** Steven S Coughlin

**Affiliations:** ^1^SelfMemphis, TNUnited States

**Keywords:** African Americans, continuing professional education, health promotion campaigns, hepatitis C

## Abstract

**Background:**

Improved drugs have been approved for the treatment of hepatitis C virus (HCV), but many people are unaware of improved therapies that are now available to cure the illness in a high percentage of patients.

**Objective:**

The objectives of the Test, Listen, Cure (TLC) Hepatitis C Community Awareness Campaign include the development and implementation of a health education and promotion campaign in Memphis, Tennessee, and surrounding areas of western Tennessee, eastern Arkansas, and northern Mississippi, to increase community awareness about HCV, and to provide up-to-date provider education on HCV screening and treatment. The health education and promotion campaign, which will be conducted in collaboration with area hospitals, clinics, and nonprofit organizations, will provide information about how HCV infection is transmitted, risk factors for the disease, the importance of screening and treatment, and the availability of improved treatment for the disease. A second objective will be to provide continuing professional education on HCV screening and treatment to a minimum of 200 area health care providers, including primary care and internal medicine physicians and residents, physician assistants, nurse practitioners, providers who care for homeless persons, and dialysis unit nurses.

**Methods:**

Health education materials will be developed for this community awareness campaign that is culturally appropriate for African Americans and suitable for people with lower health literacy and educational attainment. Information will be compiled and disseminated about area providers who provide screening services and treatment for persons with HCV in order to facilitate linkages to care. Four focus groups of 8-10, African American adults aged 40-64, will be conducted to test the health education materials. The provider education on HCV will also address patient-physician communication and cultural competency. The National Medical Association regional chapters and expert physician consultants will provide assistance with delivering the education program.

**Results:**

Results from this one year project will be available in early 2016.

**Conclusions:**

Depending on the availability of funding and successful implementation of the project, the TLC campaign will be extended to similar cities in the United States.

## Introduction

### Background

Hepatitis C, a leading cause of liver failure and liver cancer, is more common among at-risk populations including African Americans [1]. Many people who are infected with hepatitis C virus (HCV) are unaware that they have the viral infection. Until this year, treatments for HCV were interferon-based and included the antiviral ribavirin, which have several unpleasant and potentially serious side effects including depression and anemia. Many patients were unable to tolerate these side effects and patient adherence was suboptimal. In addition, therapies such as interferon combined with ribavirin were not as effective in achieving sustained remission in patients with genotype 1 HCV, which is more common among African Americans.

The Food and Drug Administration (FDA) recently approved new drugs for the treatment of HCV. These and other drugs are revolutionizing the treatment of this illness [2,3]. Interferon-free drug regimens for the treatment of HCV will likely become available for routine use by the end of 2014. However, many people are unaware of improved therapies that are now available to cure the illness in a high percentage of patients.

The overarching goal of the TLC Hepatitis C Community Awareness Campaign is to work with a community coalition of hospitals, clinics, and nonprofit organizations to raise awareness among adults and health care providers in Memphis, Tennessee, and similar cities in the United States, about the desirability of HCV screening and the availability of improved therapies for the disease. The materials developed for this community awareness health education and promotion campaign, which will be carefully evaluated, will be culturally appropriate for African Americans living in Memphis and similar localities in the United States. A further goal will be to provide up-to-date continuing professional education on HCV screening and treatment to area health care providers, including primary care and internal medicine physicians and residents, physician assistants, nurse practitioners, providers who care for homeless persons, and dialysis unit nurses. The provider education on HCV will also address patient-physician communication and cultural awareness.

### Objectives

The objectives of this project include: (1) the development and implementation of a consumer health education and promotion campaign in Memphis, Tennessee to increase community awareness about HCV, (2) the provision of up-to-date provider education on HCV screening and treatment, and (3) the extension of the project to other cities in the United States that have sizeable African American populations. The consumer health education and promotion campaign will be conducted in collaboration with area hospitals, clinics, and nonprofit community-based organizations. It will provide information about how HCV infection is transmitted, risk factors for the disease, the importance of screening and treatment, and the availability of improved treatment for the disease. Consumer health education and promotion materials will be tailored for the community awareness campaign, which is culturally appropriate for African Americans and suitable for people with lower health literacy and educational attainment. Information will be compiled and disseminated about area providers who provide screening services and treatment for persons with HCV in order to facilitate linkages to care. Continuing professional education on HCV screening and treatment will be provided to area health care providers, including primary care and internal medicine physicians, physician assistants, nurse practitioners, providers who care for homeless persons, and dialysis unit nurses. Provider education on HCV also will address patient-physician communication and cultural competency.

### Rationale for the Program

HCV is the most common chronic blood-borne pathogen in the United States [4]. Among noninstitutionalized people in the United Sates, the prevalence of HCV antibody is about 1.6%. There are an estimated 2.7 to 3.9 million individuals living with HCV in the United Sates [5]. HCV infection is about four times as common as HIV infection [6]. Persons born during 1945-1965 account for about 3/4 of all HCV infections in the United Sates. Many people who are infected with HCV (about 75%) are unaware that they have the viral infection [7]. Most patients do not experience any symptoms such as fatigue, fever, loss of appetite, nausea, vomiting, abdominal pain, joint pain, and jaundice [8]. Chronic HCV infection occurs in about 78% of infected patients [4]. About 7% to 24% of persons with chronic HCV infection develop cirrhosis after a period of 20 years [9]. The development of cirrhosis is hastened by increased alcohol consumption [7]. HCV-related end-stage liver disease is the most common indication for liver transplantation among adults in the United Sates [8]. The annual cost of untreated HCV in the United States has been estimated to be $5.5 billion [10]. Risk factors for HCV infection include past or present injection drug use, sex with an injection drug user, blood transfusion before 1992, long-term hemodialysis, being born to an HCV-infected mother, incarceration, intranasal drug use, getting an unregulated tattoo, and other percutaneous exposures such as in health care workers [4]. In the presence of maternal HCV viremia, risk for maternal-to-child transmission is about 4% to 7%, and the risk for HCV transmission is even higher when the mother has both HCV and HIV infection [11]. Remote or long-term injection drug use is the most common risk factor for HCV infection.

The first-generation protease inhibitors approved by the Food and Drug Administration (FDA) for the treatment of genotype 1 HCV infection, telaprevir and boceprevir, led to a sustained virologic response in 68% to 75% of treatment-naïve patients [2,3]. In January of this year, the FDA approved two new drugs, daclatasvir and sofosbuvir, for the treatment of HCV. These and other drugs are revolutionizing the treatment of this illness. Interferon-free drug regimens for the treatment of HCV will likely become available for routine use later in 2014. However, even among those who have been tested and are aware of their HCV infection, many people are unaware that improved therapies are now available to cure the illness in a high percentage of patients. There is also a need to provide continuing professional education on HCV screening and treatment to primary care physicians and other health care providers, especially educational programs that also address patient-physician communication and cultural awareness. The successful treatment of HCV infection improves health-related quality of life and reduces mortality among patients with HCV [12]. Although the advent of highly effective therapies creates unprecedented opportunities to prevent HCV transmission and disease, there remains a pressing need to address racial disparities in screening and treatment for HCV.

The Institute of Medicine [13] recommended that action be taken to address this “underappreciated health concern for the nation.” In 2011, the US Department of Health and Human Services published a viral hepatitis action plan that highlighted the need for community education, expanded access to HCV testing and treatment, and provider education [14]. The National Medical Association’s Hepatitis C Task Force concluded that “there is an urgent need for an enhanced effort to increase awareness of hepatitis in general, and HCV in particular” [7]. The consensus panel (Task Force) recommended CDC pamphlets and other educational tools to educate the public about HCV “be tested to ensure concordance with African American culture, values, and attitudes” [7]. The consensus panel noted that about 14% of Americans and 24% of African Americans are functionally illiterate. The consensus panel also recommended that training on HCV be provided to current and future health care providers. Many physicians require education on the prevention and early detection of HCV, and on the new updated treatments [7]. The United States Preventive Services Task Force (USPSTF) recommends screening for HCV infection in persons at high risk for infection [15]. The USPSTF also recommends offering one-time screening for HCV infection to adults born between 1945 and 1965 [4]. Anti-HCV antibody enzyme-linked immunosorbent assay (ELISA) testing followed by confirmatory polymerase chain reaction testing has been found to accurately detect chronic HCV infection.

Liu et al [16] examined the proportion of current, unresolved HCV infections in the United States in a population aged > 40 years based upon HCV RNA positivity and HCV antibody test results. Of 13,909 participants examined, 304 were anti-HCV-positive. Of these, 238 or 75.3% had detectable viral RNA. The percentage of current, unresolved HCV infection was highest among nonHispanic blacks (91.1%).

As part of planning an education campaign to raise awareness about viral hepatitis in the United Sates, the Centers for Disease Control and Prevention conducted 16 focus groups involving a total of 119 adults aged 35 to 60 years in Boston, Chicago, and Houston [17]. Awareness and knowledge of viral hepatitis were low among all participants. Little was known about different types of hepatitis, risk factors, or how the viruses are transmitted. Many participants assumed that if they had viral hepatitis, they would have symptoms and knew they were infected [17]. The authors concluded that their findings indicate that significant and concerted educational efforts are needed to improve basic knowledge of viral hepatitis, and knowledge about transmission, risk factors, screening, and treatment. Lower educational attainment and lack of a primary care provider have been identified as barriers to HCV screening [18].

In the United Sates, those who have a low family income or were born between 1945 and 1964 have a disproportionate burden of HCV, along with Vietnam veterans and nonHispanic black males [19,20]. HCV infection is an important public health problem in many inner city neighborhoods where multiple health disparities are common, including coinfection of HCV and HIV [21,22]. Homeless adults have been identified as an at-risk population for HCV infection [6,23,24]. Strehlow et al [24] examined the prevalence, distribution, and risk factors for HCV infection among homeless adults using eight Health Care for the Homeless clinics, funded by the Bureau of Primary Health Care, United Sates Health Resources and Services Administration. Data were collected for 387 homeless participants through blood draws, chart reviews, and structured interviews. The overall prevalence of HCV-antibody positivity was 31.0%. The majority (53.3%) of HCV-antibody positive, homeless participants were unaware of their status [24]. Gelberg et al [6] identified a community-based probability sample of 534 homeless adults from 41 shelters and meal programs in the skid row area of downtown Los Angeles, California. About 26.7% of the sample tested HCV-positive and 4.0% tested HIV-positive. In logistic regression analysis, independent predictors of HCV infection included older age, less education, prison history, and history of drug injection. Among HCV-infected adults, nearly half (46.1%) were unaware of their infection. Few had received any HCV-related treatment [6].

### Black-White Disparities in HCV

African Americans had the highest mortality rates from HCV in the United Sates from 2004 to 2008, at 6.5 to 7.8 deaths per 100,000 persons and died from HCV 78.9% more often than whites [25]. This disparity in HCV mortality rates increased between 2008 to 2010 [7]. African Americans also are over-represented among newly reported cases of HCV [1,7]. National data on the prevalence of HCV among African Americans and other racial/ethnic groups are limited by the lack of inclusion of homeless populations and incarcerated persons. Incidence data based upon reported cases from the CDC Viral Hepatitis Surveillance System are limited by the failure to capture race/ethnicity for more than 50% of the cases [7]. Among US veterans who had Department of Veterans Affairs (VA) outpatient visits in 2011, 53.4% underwent HCV screening [26]. Among male veterans born from 1945 through 1965, the prevalence of HCV infection was 18.2%. The prevalence of HCV infection was highest among black veterans (12.3%). Tohme et al [27] examined the rates and determinants of HCV testing, infection, and linkage to care among US racial/ethnic minorities using data from the 2009-2010 Racial and Ethnic Approaches to Community Health Across the US Risk Factor Survey (n=53,896 minority adults). Overall, only 19% of respondents were tested for HCV, including about 60% of those reporting a risk factor. College-educated, nonHispanic blacks and Asians had lower odds of HCV infection than those who did not finish high school. Among those who were infected, 44.4% were currently being followed by a physician and 41.9% had taken HCV medications. The authors concluded that HCV testing and linkage to care among racial/ethnic minorities in the United Sates are suboptimal and that further HCV testing and prevention activities should be directed toward racial/ethnic minorities, especially those of low socioeconomic status [27]. Trooskin et al [28] studied 4407 charts from four primary care sites, two community clinics, and two academic, primary care practices in Philadelphia. They found that African Americans were less likely to be referred to a subspecialist for treatment when they tested positive for HCV infection [28]. The National Medical Association’s Hepatitis C Task Force found that many of the risk factors associated with HCV prevalence and incidence are socially determined [7]. Black-white disparities in HCV treatment outcomes and progression to liver disease and/or primary hepatocellular carcinoma also have been reported [7]. African Americans are more likely to have advanced HCV-related tumor stage at diagnosis and less likely to receive local or surgical therapy than whites, even with tumors that are localized to the liver.

Other studies have shown that effective patient-physician communication is related to improved adherence to medical regimens, better decision making, and increased satisfaction with the patient-physician relationship [29,30]. Cultural competency skills can assist patient-provider communication. Cultural competency influences how health messages are transmitted and perceived, how illness is defined, how symptoms are described, when and where care is obtained, and how treatment options are considered. Cultural competency includes the acquisition and integration of knowledge, with awareness, attitude, and skills about culture and cultural differences that enables health care professionals to provide optimal care to patients from different racial, ethnic, socioeconomic, and cultural backgrounds [31].

Patient health literacy is also important. Low health literacy has been associated with decreased use of preventive services such as screening tests, increased risk of having a chronic disease, increased use of emergency services, poorer treatment adherence, and poorer health outcomes [32]. The Institute of Medicine defines health literacy as “the degree to which individuals have the capacity to obtain, process, and understand basic health information and services needed to make appropriate health decisions” [33]. Disease prevention and treatment messages are often written at too high a reading level for individuals with marginal literacy skills.

## Methods

### The Campaign

To increase community awareness and encourage at-risk African American residents to be screened for HCV, a coordinated community-based health education and promotion campaign will be conducted in collaboration with the Shelby County Health Department, the Regional One Health, the University of Tennessee Medical Group, National Medical Association regional chapters, and local nonprofit organizations. The campaign will maximize media attention on HCV infection, screening, and treatment beginning in the second quarter of year one. Representatives will be invited from the local radio, television, print and social media to participate and assist with the dissemination of information. The campaign will strive to ensure that all African American adults in Memphis, and other adults in the mid-south, are exposed to multiple messages about risk factors for HCV infection, HCV screening, and the availability of improved treatments for the disease. In addition to newspaper and OpEd articles and radio and television appearances and public service announcements, pamphlets and posters (small media) will be distributed at familiar community sites such as churches, markets, clinics, barbershops, hair salons, and laundromats. The identification of educational materials will be facilitated by resources distributed by the CDC Division of Viral Hepatitis website. Each material will be evaluated for readability and cultural sensitivity with the assistance of focus groups comprised of 8 to 12 African American men and women ages 40 to 70 years, recruited through collaborating hospitals and clinics. IRB approval will be obtained. Health literacy readability will be measured using the short form of the Test of Functional Health Literacy in Adults (S-TOFHLA) which takes 12 minutes to administer and consists of reading comprehension (2 passages) and numeracy (5 questions) sections. The Lipkus numeracy scale will be used to assess numeracy. The scale is comprised of general numeracy items and health specific numeracy items. Questions are scored as percent correct. The Cultural Sensitivity Assessment Tool will also be used in the focus groups to evaluate the cultural sensitivity of educational materials for African American adults. Scores range from 4 to 1 with scores < 50 indicating print materials are culturally insensitive. The theoretical theories or constructs will include the Health Belief Model, Social Ecological Theory, and social marketing techniques. Public service announcements and the importance of HCV screening and treatment will be disseminated through radio stations that target African American adults in Memphis and surrounding areas of the mid-south. The desirability of using black radio to disseminate health messages to the African American community and reduce health disparities has recently been highlighted [34]. Black radio has advantages over print media for circumventing low health literacy. There are several newspapers in Memphis and the mid-south region that belong to the National Newspaper Publishers Association, a black community newspaper organization, as shown in Table 1.

**Table 1:** Black community newspapers in the mid-south, United States.

Memphis Silver Star News	3019 Park Ave Memphis, Tennessee 38114 Phone: 901-452-8828 Email: silverstarnews@bellsouth.net
Nashville Pride	PRIDE Publishing Group 315 Deaderick Street,Suite 1575 Nashville,TN 37238 Phone: 615-292-9150
The Tennessee Tribune	1501 Jefferson St Nashville, TN 37208 Phone: 615-321-3268 Email: info@tntribune.com
Tri-State Defender	203 Beale Street, Suite 200 Memphis Tennessee 38103 Phone: 901-523-1818 Email: besmith@tri-statedefender.com

An innovative professional continuing education program will be developed and offered on three occasions to interested health care providers from Memphis and surrounding areas of the mid-south. Physicians and other providers will receive letters inviting them to participate with the assistance of CEOs, medical directors, and regional chapters of the National Medical Association. The curriculum for the educational program will be adapted from educational materials developed by the National Medical Association and other leading professional societies. The educational materials will be informed by the USPSHS guidelines for screening for HCV. The learning objectives will be carefully specified. For example, at the end of the session, participants should be able to demonstrate usage of the U.S. Preventive Health Services Task Force (USPHSTF) guidelines, list methods of incorporating the PHS guidelines into their practices, and identify resources for educating their patients about HCV treatment options. Cultural competency and patient-provider communication training will be provided and the participating providers will be given opportunities to practice these skills in the context of providing HCV treatment. The duration of the continuing professional education will be limited to two, half-day, participatory sessions located at convenient community hospitals and clinics. The training program will be offered on three occasions with the assistance of National Medical Association regional chapters.

The participating health care providers will be asked to fill out self-administered questionnaires about their professional background and medical practice. Both pre- and post-educational intervention questionnaires will be administered so that their experience and confidence in providing HCV screening can be assessed, along with their knowledge and attitude about HCV treatment advances. Several questions will be included in the post-intervention questionnaire so as to allow the participating physicians to help evaluate the continuing education program and suggest future refinements.

### Target Populations

The professional education program on HCV screening and treatment will target interested health care providers from Memphis and surrounding areas of the mid-south (primary care and internal medicine physicians and residents, physician assistants, nurse practitioners, providers who care for homeless persons, and dialysis unit nurses). These providers will be identified with the assistance of the University of Tennessee Medical Group, Methodist-Le Bonheur Healthcare, the Med Regional Medical Clinic, regional chapters of the National Medical Association, and other community and professional organizations.

The target population for the community awareness campaign consists of Memphis and surrounding areas of western Tennessee, eastern Arkansas, and northern Mississippi. Memphis has a population of 662,897 of which 419,614 (63.3%) are African Americans compared to 16.7% for the state. The poverty rate in Memphis is 27.2% and the overall poverty rate for the working poor is 49.3%. Of all households, 47.1% are headed by a single female. African Americans are less likely to graduate from high school and African American adults are less likely to have a college degree. Social determinants such as poverty, lower educational attainment, and unemployment are significant barriers and challenges to receipt of health care for many African Americans in Memphis and other US cities. In 2011, the rate of reported cases of acute HCV infection in Tennessee was 1.3 per 100,000 population, a rate second only to Oklahoma and Kentucky, although these data are limited by incomplete reporting in some states [7].

### Plan for Evaluation

The implementation of the community awareness campaign will be monitored through process evaluation measures such as the number of pamphlets and brochures distributed at community sites, the number, date, and location of newspaper articles about HCV, and the number and date of radio public service announcements.

The health care providers who participate in the continuing professional education program will be asked to fill out self-administered questionnaires about their professional background and medical practice. Both pre- and post-educational intervention questionnaires will be administered so that their experience and confidence in providing HCV screening can be assessed, along with their knowledge and attitudes about HCV treatment advances.

### Data Analysis Plan

Cross-tabulations of the data will be performed to analyze process evaluation data from the community awareness campaign and information collected as part of the continuing professional education program for health care providers.

## Results

It is anticipated that results from this one year project will be available in early 2016.

## Discussion

 Depending on the availability of funding and successful implementation of the project, the TLC campaign will be extended to similar cities in the United States. The advent of oral drug regimens for hepatitis C has increased the feasibility of increased population screening and successful treatment. Although the high cost of treatment has been an important barrier for many patients, the recent introduction of additional FDA-approved oral combination therapies has led to competition between manufacturers and some price concessions. There is a need for additional efforts to provide continuing professional education about these important developments and to increase community awareness about the availability of improved therapies for hepatitis C.

## Abbreviations

CDC: Centers for Disease Control and Prevention
FDA: Food and Drug Administration
ELISA: enzyme-linked immunosorbent assay
HCV: hepatitis C virus
RNA: ribonucleic acid
USPHSTF: United States Preventive Health Services Task Force
TOFHLA: test of functional health literacy in adults
VA: Veterans Affairs

## References

[1] Bailey RK, Muir AJ, Howell CD, Bright C, Roane PR, Teshale E, Johnson CM, Curry SB, Collins AC, Jordan W, Allison-Ottey SD (2013). The hepatitis C crisis in the African American Community: findings and recommendations. J Natl Med Assoc.

[2] Poordad F, McCone J, Bacon BR, Bruno S, Manns MP, Sulkowski MS, Jacobson IM, Reddy KR, Goodman ZD, Boparai N, DiNubile MJ, Sniukiene V, Brass CA, Albrecht JK, Bronowicki JP, SPRINT-2 Investigators (2011). Boceprevir for untreated chronic HCV genotype 1 infection. N Engl J Med.

[3] Jacobson IM, McHutchison JG, Dusheiko G, Di Bisceglie AM, Reddy KR, Bzowej NH, Marcellin P, Muir AJ, Ferenci P, Flisiak R, George J, Rizzetto M, Shouval D, Sola R, Terg RA, Yoshida EM, Adda N, Bengtsson L, Sankoh AJ, Kieffer TL, George S, Kauffman RS, Zeuzem S, ADVANCE Study Team (2011). Telaprevir for previously untreated chronic hepatitis C virus infection. N Engl J Med.

[4] Moyer VA, U.S. Preventive Services Task Force (2013). Screening for hepatitis C virus infection in adults: U.S. Preventive Services Task Force recommendation statement. Ann Intern Med.

[5] Smith BD, Morgan RL, Beckett GA, Falck-Ytter Y, Holtzman D, Teo CG, Jewett A, Baack B, Rein DB, Patel N, Alter M, Yartel A, Ward JW, Centers for Disease ControlPrevention (2012). Recommendations for the identification of chronic hepatitis C virus infection among persons born during 1945-1965. MMWR Recomm Rep.

[6] Gelberg L, Robertson MJ, Arangua L, Leake BD, Sumner G, Moe A, Andersen RM, Morgenstern H, Nyamathi A (2012). Prevalence, distribution, and correlates of hepatitis C virus infection among homeless adults in Los Angeles. Public Health Rep.

[7] Bailey RK, Bright C, Howell CD, Muir AJ, Curry SB, Mouton CP, Roane PR (2013). Hepatitis C: A crisis in the African American community. Findings and recommendations.

[8] Fusfeld L, Aggarwal J, Dougher C, Vera-Llonch M, Bubb S, Donepudi M, Goss TF (2013). Assessment of motivating factors associated with the initiation and completion of treatment for chronic hepatitis C virus (HCV) infection. BMC Infect Dis.

[9] Kanda T, Imazeki F, Yokosuka O (2010). New antiviral therapies for chronic hepatitis C. Hepatol Int.

[10] Leigh JP, Bowlus CL, Leistikow BN, Schenker M (2001). Costs of hepatitis C. Arch Intern Med.

[11] Jou JH, Muir AJ (2012). Hepatitis C. Ann Int Med.

[12] Sussman NL, Remien CH, Kanwal F (2014). The end of hepatitis C. Clin Gastroenterol Hepatol.

[13] Colvin Hm, Mitchell AE (2010). Hepatitis and liver cancer: a national strategy for prevention and control of hepatitis B and C.

[14] Ward JW, Valdiserri RO, Koh HK (2012). Hepatitis C virus prevention, care, and treatment: from policy to practice. Clin Infect Dis.

[15] U.S. Preventive Services Task Force.

[16] Liu G, Holmberg SD, Kamili S, Xu F (2014). Racial disparities in the proportion of current, unresolved hepatitis C virus infections in the United States, 2003-2010. Dig Dis Sci.

[17] Jorgensen CM, Carnes CA (2013). Lessons learned from exploratory research about viral hepatitis. Health Promot Pract.

[18] Barocas JA, Brennan MB, Hull SJ, Stokes S, Fangman JJ, Westergaard RP (2014). Barriers and facilitators of hepatitis C screening among people who inject drugs: a multi-city, mixed-methods study. Harm Reduct J.

[19] Armstrong GL, Wasley A, Simard EP, McQuillan GM, Kuhnert WL, Alter MJ (2006). The prevalence of hepatitis C virus infection in the United States, 1999 through 2002. Ann Intern Med.

[20] Kuehn BM (2009). Silent epidemic of viral hepatitis may lead to boom in serious liver disease. JAMA.

[21] Coughlin SS (2011). Invited commentary: co-occurring health conditions among women living with profound life challenges. Am J Epidemiol.

[22] Searson G, Engelson ES, Carriero D, Kotler DP (2014). Treatment of chronic hepatitis C virus infection in the United States: some remaining obstacles. Liver Int.

[23] Hall CS, Charlebois ED, Hahn JA, Moss AR, Bangsberg DR (2004). Hepatitis C virus infection in San Francisco's HIV-infected urban poor. J Gen Intern Med.

[24] Strehlow AJ, Robertson MJ, Zerger S, Rongey C, Arangua L, Farrell E, O'Sullivan A, Gelberg L (2012). Hepatitis C among clients of health care for the homeless primary care clinics. J Health Care Poor Underserved.

[25] (2010). Centers for Disease Control.

[26] Backus LI, Belperio PS, Loomis TP, Yip Gh, Mole La (2013). Hepatitis C virus screening and prevalence among US veterans in Department of Veterans Affairs care. JAMA Intern Med.

[27] Tohme RA, Xing J, Liao Y, Holmberg SD (2013). Hepatitis C testing, infection, and linkage to care among racial and ethnic minorities in the United States, 2009-2010. Am J Public Health.

[28] Trooskin SB, Navarro VJ, Winn RJ, Axelrod DJ, McNeal AS, Velez M, Herrine SK, Rossi S (2007). Hepatitis C risk assessment, testing and referral for treatment in urban primary care: role of race and ethnicity. World J Gastroenterol.

[29] Makoul G, Curry RH (2007). The value of assessing and addressing communication skills. JAMA.

[30] Diefenbach M, Turner G, Carpenter KM, Sheldon LK, Mustian KM, Gerend MA, Rini C, von Wagner C, Gritz ER, McQueen A, Prayor-Patterson HM, Miller SM (2009). Cancer and patient-physician communication. J Health Commun.

[31] Kagawa-Singer M, Dadia AV, Yu Mc, Surbone A (2010). Cancer, culture, and health disparities: time to chart a new course?. CA Cancer J Clin.

[32] Berkman ND, Sheridan SL, Donahue KE, Halpern DJ, Crotty K (2011). Low health literacy and health outcomes: an updated systematic review. Ann Intern Med.

[33] Nielsen-Bohlman L, Panzer AM, Hamlin B, Kindig DA (2004). Health literacy: a prescription to end the confusion.

[34] Hall IJ, Johnson-Turbes A, Williams KN (2010). The potential of black radio to disseminate health messages and reduce disparities. Prev Chronic Dis.

